# A novel role for Helicobacter pylori cytotoxin-associated gene A in negative regulation of autophagy in human gastric cells

**DOI:** 10.1186/s12876-023-02944-8

**Published:** 2023-09-22

**Authors:** Akihiko Sakatani, Yoshito Hayashi, Hirotsugu Saiki, Minoru Kato, Ryotaro Uema, Takanori Inoue, Keiichi Kimura, Shunsuke Yoshii, Yoshiki Tsujii, Shinichiro Shinzaki, Hideki Iijima, Tetsuo Takehara

**Affiliations:** Department of Gastroenterology and Hepatology, Osaka University Graduate School of Medicine, 2-2 Yamadaoka, Suita, Osaka Japan

**Keywords:** *Helicobacter pylori*, Cytotoxin-associated gene A, Autophagy, Gastric cancer

## Abstract

**Background:**

Autophagy plays an important role in carcinogenesis and tumor progression in many cancers, including gastric cancer. Cytotoxin-associated gene A (CagA) is a well-known virulent factor in *Helicobacter pylori* (*H. pylori*) infection that plays a critical role in gastric inflammation and gastric cancer development. However, its role in autophagy during these processes remains unclear. Therefore, we aimed to clarify the role of CagA in autophagy in CagA-related inflammation.

**Methods:**

We evaluated the autophagic index of AGS cells infected with wild-type *cagA-*positive *H. pylori* (*Hp-WT*) and *cagA*-knockout *H. pylori* (*Hp-*Δ*cagA*) and rat gastric mucosal (RGM1) cells transfected with CagA genes. To identify the mechanisms underlying the down regulation of autophagy in AGS cells infected with *H. pylori*, we evaluated protein and mRNA expression levels of autophagy core proteins using western blotting and quantitative reverse transcription-polymerase chain reaction (RT-PCR). To determine whether autophagy induced the expression of the pro-inflammatory mediator, cyclooxygenase-2 (COX-2), we evaluated COX-2 expression in AGS cells treated with an autophagy inducer and inhibitor and infected with *H. pylori*. In addition, we evaluated whether COX-2 protein expression in AGS cells influenced beclin-1 (BECN1) expression with si-RNA transfection when infected with *H. pylori*.

**Results:**

Autophagic flux assay using chloroquine showed that autophagy in AGS cells was significantly suppressed after *H. pylori* infection. The autophagic index of AGS cells infected with *Hp-WT* was decreased significantly when compared with that in AGS cells infected with *Hp-*Δ*cagA*. The autophagic index of RGM1 cells transfected with CagA was lower, suggesting that CagA inhibits autophagy. In addition, BECN1 expression levels in AGS cells infected with *Hp-WT* were reduced compared to those in AGS cells infected with *Hp-*Δ*cagA*. Furthermore, COX-2 expression in AGS cells infected with *H. pylori* was controlled in an autophagy-dependent manner. When AGS cells were transfected with small interfering RNA specific for BECN1 and infected with *Hp-WT* and *Hp-*Δ*cagA,* COX-2 was upregulated significantly in cells infected with *Hp-*Δ*cagA*.

**Conclusions:**

In conclusion, the *H. pylori* CagA protein negatively regulated autophagy by downregulating BECN1. CagA-induced autophagy inhibition may be a causative factor in promoting pro-inflammatory mediator production in human gastric epithelial cells*.*

**Supplementary Information:**

The online version contains supplementary material available at 10.1186/s12876-023-02944-8.

## Background

*Helicobacter pylori* is a gram-negative, spiral-shaped bacterium that colonizes the human gastric mucosa. *H. pylori* infection leads to the development of chronic gastritis, ulcers, gastric intestinal metaplasia, dysplasia, and subsequent adenocarcinoma. Several virulence factors produced by *H*. *pylori* contribute to its pathogenesis. Cytotoxin-associated gene A (CagA) and vacuolating cytotoxin A (VacA) are the most important virulence factors associated with an increased risk of inflammation and carcinogenesis [1]. CagA is one of the major bacterial oncoprotein delivered into gastric epithelial cells through a type IV secretion system and which undergoes tyrosine phosphorylation and activates a series of intracellular signal transduction reactions, resulting in tissue inflammation [2]. A previous study has reported that functional crosstalk between CagA and VacA enhances the viability of *H. pylori* in the hostile gastric environment, limiting cellular damage, and improving infection efficiency [3].

Autophagy is an intracellular degradation process involving the combination of autophagosomes and lysosomes, which contain degradative enzymes [4]. It is induced by cellular stresses produced during starvation, DNA damage, reactive oxygen species accumulation, and the presence of damaged organelles and invading microbes [4, 5]. Aggregated proteins and organelles are sequestered by double-membraned autophagosomes, which subsequently fuse with lysosomes to mediate breakdown. The degradation sequence above is called autophagic flux. It can be measured using various methods, and it is used as an indicator of autophagic degradation activity. Autophagy maintains cellular and tissue homeostasis by removing damaged or unwanted organelles and proteins, recycling materials, and producing energy, and its dysregulation is involved in the pathogenesis of various diseases in humans, [6] including gastric cancer induced via several different mechanisms [7–12].

In recent years, *H. pylori* infection has been shown to modulate the autophagy pathway in the host [8–11]. Raju et al. reported that prolonged exposure of human gastric epithelial cells to VacA disrupts autophagy induction [9]. Tsugawa et al. have demonstrated that intracellular CagA is degraded by an autophagy-dependent mechanism [10]. They reported that the binding of m1VacA to low-density lipoprotein receptor 1 is required for reducing intracellular glutathione levels and inducing autophagy, resulting in CagA degradation. Although several reports indicate that VacA functions as the virulence factor that mediates regulation of autophagy in human gastric epithelial cells infected with *H. pylori* [8, 9], the role of CagA in autophagy has not been investigated extensively. Recently, Li et al. demonstrated that CagA inhibits autophagy via the c-Met-PI3K/Akt-mTOR signaling pathway [13]. However, the effect of CagA on the autophagic machinery core protein associated with autophagosome formation was not assessed in their studies, although they demonstrated that CagA reduces the number of autophagic vacuoles and inhibits autophagy. Hence, we hypothesized that an additional pathway apart from the c-Met-PI3K/Akt-mTOR signaling pathway may be regulated by CagA in human gastric epithelial cells infected with *H. pylori*. Moreover, Li et al. demonstrated that autophagy plays a role in the expression of proinflammatory cytokines such as interleukin 8 (IL-8), IL-1β, and tumor necrosis factor alpha (TNF-α) [13]. However, the effect of autophagy on the production of cyclooxygenase 2 (COX-2), an enzyme involved in prostanoid synthesis and the development and progression of gastric cancer, is unclear.

In the present study, the effect of CagA on autophagy in gastric epithelial cells and the production of autophagy-regulated pro-inflammatory mediator were determined, while focusing on COX-2.

## Methods

### Cell culture and *H. pylori* strain culture

The human gastric cancer cell line AGS was purchased from American Type Culture Collection (ATCC; Manassas, VA, USA) and cultured in RPMI 1640 medium (Gibco, New York, NY, USA, #11,765–054) supplemented with 10% fetal bovine serum (FBS; Gibco, #10,099–141) in a humidified incubator (5% CO_2_) at 37 °C. Rapamycin (100 nM, Adipogen Life Sciences, Inc., San Diego, USA, #53,123–88-9) was used to induce autophagy, whereas chloroquine (20 µM, Sigma–Aldrich, Ann Arbor, MI, USA) were used to inhibit autophagy.

The wild-type *cagA*-positive *H. pylori* strain, NCTC11637 (*Hp-WT*, ATCC), and the *cagA*-knockout *H. pylori* strain with NCTC11637 background (*Hp*-Δ*cagA*) that were kindly provided by Dr. Sasakawa [14, 15] were cultured on trypticase soy agar plates (Becton Dickinson, San Diego, CA, USA) in a humidified incubator (5% CO_2_) at 37 °C.

### Preparation of CagA-expressing cells

Rat gastric mucosal cells (RGM1) were obtained from Riken Cell Bank (Tsukuba, Japan) and maintained in a Dulbecco’s Modified Eagle Medium containing 10% fetal bovine serum. Cell clones that were transfected with *CagA* under the Tet-Off system were designated as RGM1-CagA cells and those transfected with only the Tet-off system were designated as RGM1-Mock cells, as previously described [16]. RGM1 cells were maintained in the medium without tetracycline.

### Small interfering RNA (si-RNA) transfection

siRNAs against human autophagy-related 5 (*ATG5*) (s18160) and *BECN1* (s16539) along with negative control si-RNA (s18160) were purchased from Thermo Fisher Scientific (Waltham, MA, USA). siRNAs were transfected using Lipofectamine RNAiMAX (Thermo Fisher Scientific) as described in the manufacturer’s protocol.

### *H. pylori* infection

*H. pylori* was cultured for 72 h and then transferred to Brucella broth (Becton Dickinson, Sparks, MD) containing 10% FBS. After centrifuging the broth at 4,500 × *g* for 1 min, *H. pylori* was resuspended in serum-free RPMI 1640. AGS cells were infected with *Hp-WT* and *Hp-ΔcagA* (multiplicity of infection = 100:1).

### Western blotting

Western blotting was performed as previously described [17]. Blocked membranes were incubated overnight with antibodies against beta-actin (#4970), ATG5 (#2630), ATG7 (#2631), BECN1 (#3495), microtubule-associated proteins 1A/1B light chain 3A (LC3; #2775), P62(#5114), ULK1 (#6439) (Cell Signaling Technology, Danvers, MA, USA), and phospho-tyrosine (05–1050; Sigma–Aldrich, Ann Arbor, MI, USA) for CagA detection [14] at dilutions of 1:100–1:1000. All target protein expression levels were calculated and normalized to the quantified beta-actin protein expression level using ImageJ software 1.8.0_172 (https://imagej.nih.gov/). The blots in our figures were cut and cropped. Gels in the images retain important bands.

### Quantitative reverse transcription-polymerase chain reaction (RT-PCR)

Intracellular total RNA was prepared using the RNeasy Mini kit (74,106; Qiagen, Hilden, Germany), and quantitative real-time RT-PCR was performed using the ReverTra Ace qPCR RT Master Mix (FSQ-201; Toyobo, Osaka, Japan) according to the manufacturer’s instructions. Quantitative real-time RT-PCR reaction was performed using a Thunderbird SYBR qPCR Mix (QPS-201; Toyobo) on a Quant Studio 6 Flex Standard Real-Time PCR system (Applied Biosystems, Foster City, CA, USA). PCR conditions are shown in Supplemental Table1. mRNA expression levels were analyzed using the following primers synthesized by Sigma-Genosys: 5′-AGCAACTCTGGATGGGATTG-3^′^ (S) and 5^′^-CACTGCAGAGGTGTTTCCAA-3^′^ (AS) for *ATG5*; 5′-ACCGTGTCACCATCCAGGAA-3^′^ (S) and 5^′^-GAAGCTGTTGGCACTTTCTGT-3^′^ (AS) for *BECN1*; AAGAAT-3^′^; 5′-GAAGCTGTTGGCACTTTCTGT-3′ (S) and 5′-CTGCAAAAGATTGTTTGGCAGA-3′ (AS) for *cagA*; 5′-TCCCTGGAGAAGAGCTACG-3′ (S) and 5′-GTAGTTTCGTGGATGCCACA-3′ (AS) for beta-actin. Target gene expression levels were normalized to those of beta-actin.

### Autophagic flux assay

Cells were treated with 20 μM chloroquine (Sigma–Aldrich), an autophagic flux inhibitor, in diluted water 2 h before protein extraction and were subjected to western blotting. The autophagic index was calculated using the following method: autophagy flux index = (LC3-II expression levels with chloroquine)/(LC3-II expression levels without chloroquine). LC3-II expression level was normalized to the beta-actin expression levels. In each experiment, the autophagic index of the control group was normalized to 1.

### Statistical analyses

Statistical analysis was performed using JMP Pro 13.0 (SAS Institute, Cary, NC). Comparisons between groups were performed using student’s *t*-test. A value of P < 0.05 was considered significant.

## Results

### Autophagy was negatively regulated in AGS cells after *H. pylori* infection

**Fig. 1:**
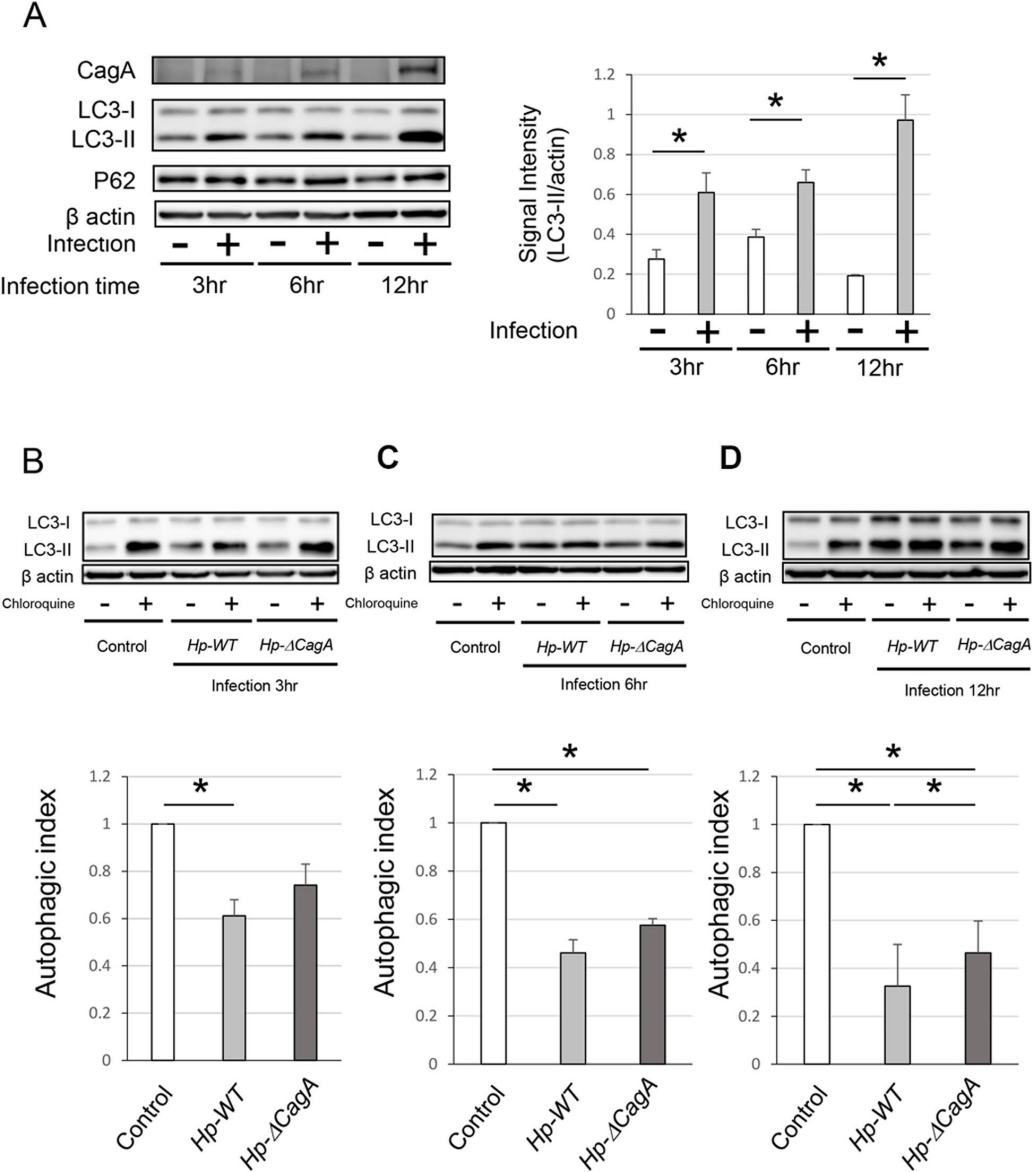
Autophagy in AGS cells was inhibited after infection with *Helicobacter pylori.* **A** Western blotting showing the protein levels of CagA, LC3, and P62 in AGS cells infected with *Hp*-*WT* for 3, 6, and 12 h (*n* = 5, mean ± SD, *P < 0.05). **B**-**D**) Western blotting and autophagic flux assays in AGS cells infected with *Hp*-*WT* and *Hp*-*ΔcagA* for 3(**B**), 6(**C**), and 12 h(**D**) (*n* = 5, mean ± SD, *P < 0.05). CagA, cytotoxin-associated gene A; LC3, microtubule-associated proteins 1A/1B light chain 3A; *Hp-WT,* wild-type *cagA-*positive *H. pylori*; *Hp-*Δ*cagA, cagA*-knockout *H. Pylori*; SD, standard deviation

To assess the effect of *H. pylori* infection on autophagy in AGS cells, the LC3 and P62 of the AGS cells infected with *Hp-WT* for 3, 6, and 12 h were measured. *Hp-WT* infection increased the protein level of LC-3II significantly (Fig. 1A). Subsequently, to validate the role of CagA in autophagy regulation, the autophagic index of AGS cells infected with *Hp-WT* or *Hp-ΔcagA* was evaluated*.* The autophagic index of AGS cells infected with *Hp-WT* for 3, 6, and 12 h and *Hp*-*ΔcagA* for 6 and 12 h (Fig. 1B-D) was decreased significantly. In addition, the autophagic index of AGS cells infected with *Hp-WT* for 12 h was decreased significantly when compared with that of AGS cells infected with *Hp-ΔcagA* (Fig. 1D). Therefore, these findings support the notion that CagA functioned as an autophagy inhibitor and modulated the autophagic flux in gastric cells infected with *H. pylori.*

### Autophagy was negatively regulated in RGM1 cells transfected with *cagA*

**Fig. 2:**
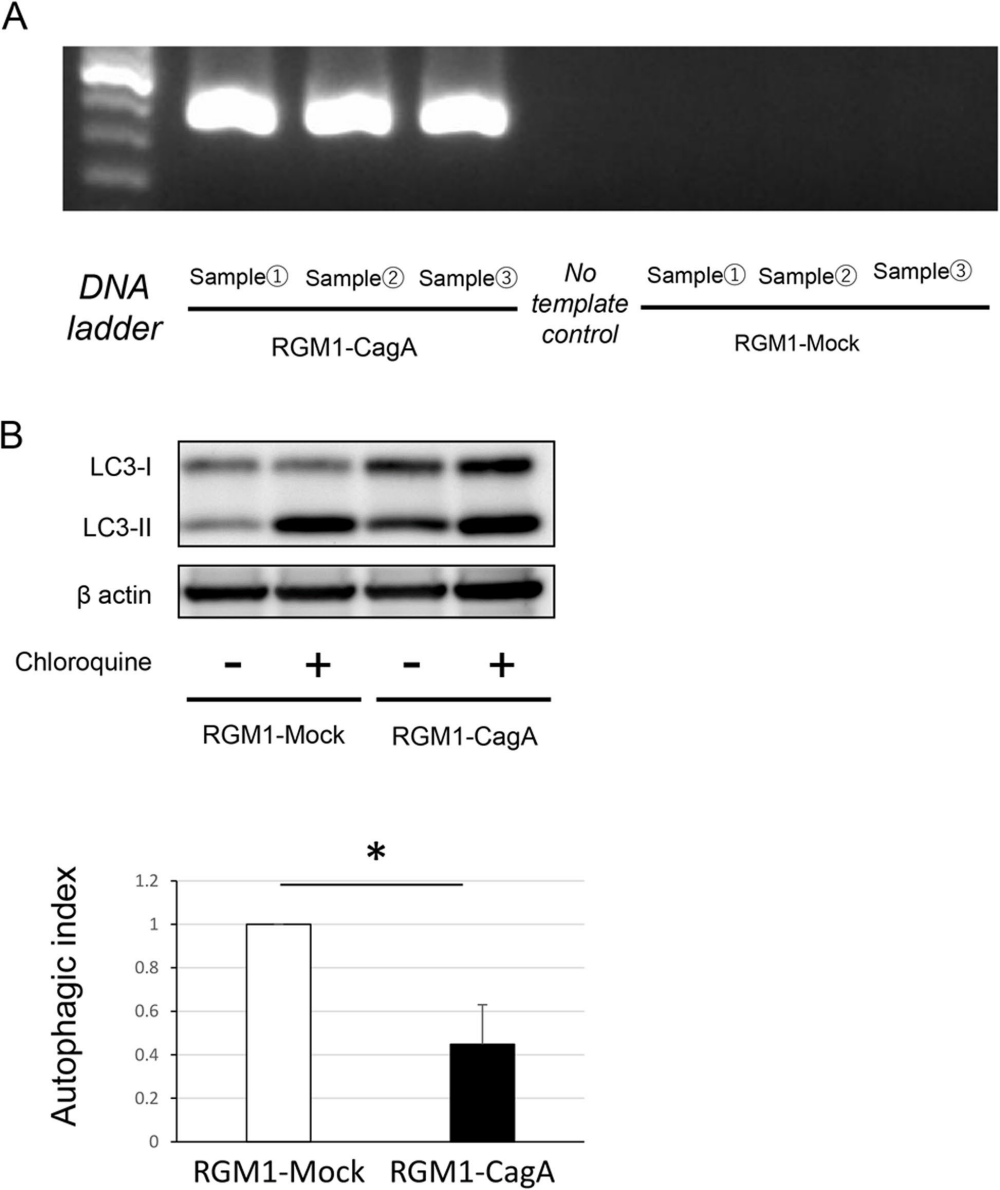
Autophagic index of RGM1 cells transfected with *cagA* was decreased compared to that of non-transfected RGM1 cells. **A** Reverse transcription-polymerase chain reaction analysis showing that *cagA* was expressed in RGM1-CagA cells. **B** Western blotting and autophagic index measurement using chloroquine (*n* = 4, mean ± SD, *P < 0.01). RGM1-CagA, RGM1 cells transfected with *cagA*; RGM1-Mock, non-transfected RGM1 cells; CagA, cytotoxin-associated gene A; SD, standard deviation

To further evaluate the effect of CagA on autophagy regulation, we evaluated the autophagic index of RGM1-Mock and RGM1-CagA cells. We first confirmed that *CagA* gene was induced constitutively in RGM1-CagA cells (Fig. 2A). The autophagic index of RGM1-CagA cells was decreased significantly when compared with that of RGM1-Mock cells (Fig. 2B). Collectively, the data suggest that intracellular CagA with or without *H. pylori* infection is an autophagy inhibitor.

### CagA negatively regulated BECN1 expression level in AGS cells infected with *H. pylori*

**Fig. 3:**
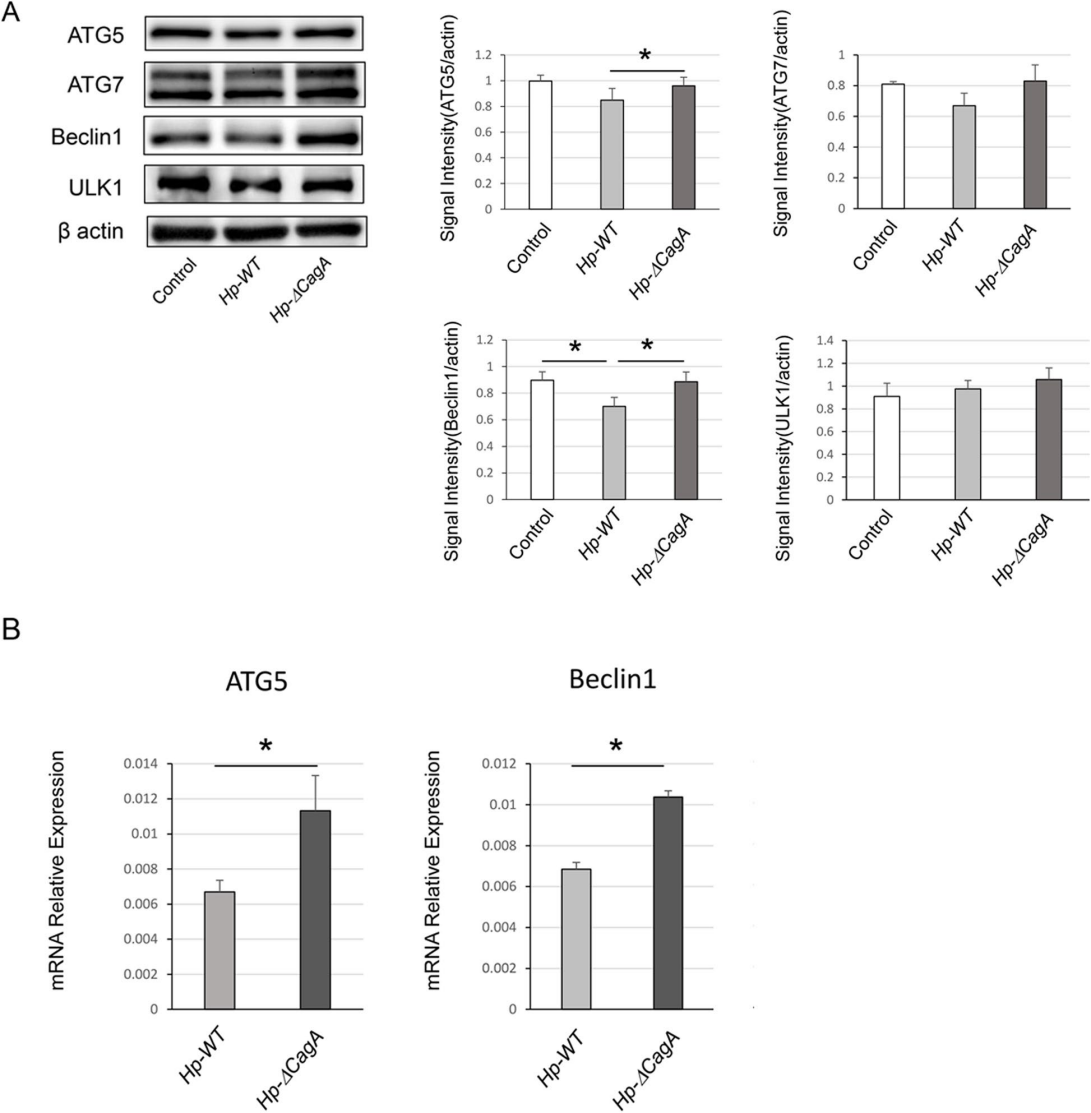
mRNA expression and protein levels of autophagic core proteins in AGS cells infected with *Helicobacter pylori*. **A** Western blotting analysis of autophagy-related proteins in AGS cells infected with *Hp-WT* and *Hp-ΔcagA.* Quantification of ATG5, ATG7, Beclin1, and ULK1 signal intensity (*n* = 4, mean ± SD, *P < 0.05). B) mRNA expression levels of *ATG5* and *BECN1* in AGS cells infected with *Hp-WT* and *Hp-ΔcagA* analysed using q-RT PCR (*n* = 6, mean ± SD, *P < 0.05). ATG5, autophagy related 5; *Hp-WT,* wild-type *cagA-*positive *H. pylori*; *Hp-*Δ*cagA, cagA*-knockout *H. Pylori*; *q-RT PCR,* quantitative reverse transcription-polymerase chain reaction; SD, standard deviation

To assess the effect of CagA on autophagic machinery core proteins associated with autophagosome formation, the protein expression levels of ATG5, ATG7, BECN1 and ULK1 were measured by western blotting (Fig. 3A). Protein expression levels of ATG5 and BECN1 in AGS cells infected with *Hp-WT* were lower than those in AGS cells infected with *Hp-ΔcagA*. No significant difference was observed in the protein expression levels of ATG7 and ULK1. In addition, mRNA expression levels of ATG5 and BECN1 in AGS cells infected with *Hp-WT* were significantly lower than those in AGS cells infected with *Hp-ΔcagA* (Fig. 3B). The data indicate that CagA downregulated *BECN1* in AGS cells infected with *H. pylori*.

### Autophagy-regulated COX-2 expression in cells after *H. pylori* infection

**Fig. 4:**
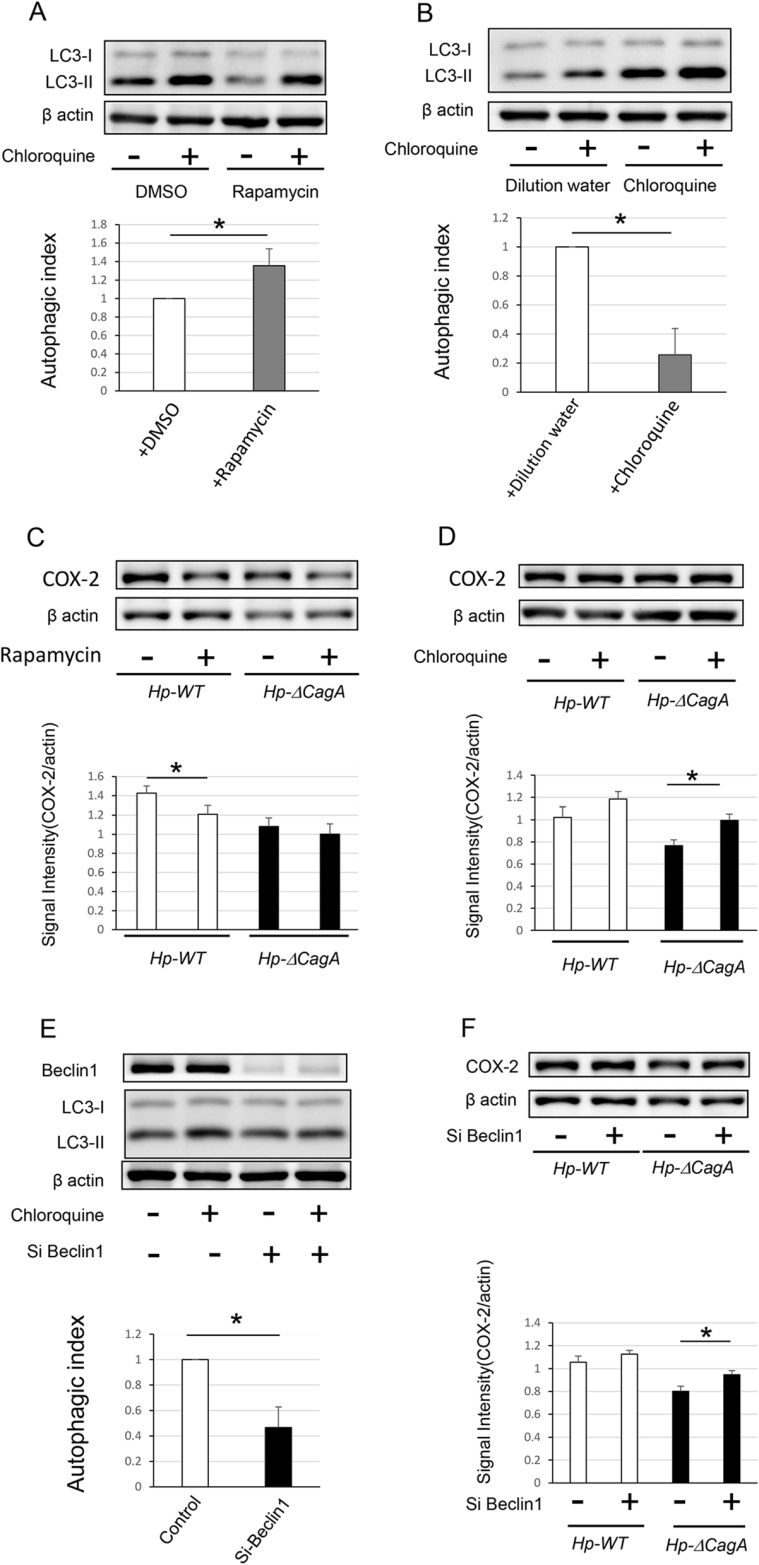
Autophagy affected protein level of COX-2 in AGS cells infected with *H. pylori.* **A** Western blotting and autophagic flux assay in AGS cells pretreated with rapamycin for 24 h (*n* = 4, mean ± SD, *P < 0.05). **B** Western blotting and autophagic flux assay in AGS cells pretreated with chloroquine for 6 h (*n* = 4, mean ± SD, *P < 0.05). **C** Western blotting and signal intensity of COX-2 protein levels in AGS cells enhanced autophagy with rapamycin infected with *Hp-WT* and *Hp-ΔcagA* (*n* = 4, mean ± SD, *P < 0.05). **D** Western blotting and signal intensity of COX-2 protein levels in AGS cells inhibited autophagy with rapamycin infected with *Hp-WT* and *Hp-ΔcagA* (*n* = 4, mean ± SD, *P < 0.05). **E** Western blotting and autophagic flux assay in AGS cells transfected with *BECN1* small interfering-RNA (*n* = 4, mean ± SD, *P < 0.05). **F** Western blotting and signal intensity of COX-2 protein levels in BECN1 knock down AGS cells infected with *Hp-WT* and *Hp-ΔcagA* (*n* = 5, mean ± SD, *P < 0.05). *Hp-WT,* wild-type *cagA-*positive *H. pylori*; *Hp-*Δ*cagA, cagA*-knockout *H. pylori*; *CagA,* cytotoxin-associated gene A; COX-2, cyclooxygenase 2

Li et al. demonstrated that levels of proinflammatory cytokines such as IL-8, TNF-α, and IL-1 and the activity of nuclear factor kappa-light-chain-enhancer of activated B cells are increased in AGS cells infected with *H. pylori* [13]. To clarify the role of CagA in inflammation and carcinogenesis, we evaluated the protein level of COX-2, which was the most-characterized enzyme in previous epidemiological and molecular studies and has a considerable association with the development and progression of gastric cancer [18]. We also examined the production of COX-2 in AGS cells treated with an autophagy enhancer (rapamycin) or inhibitor (chloroquine) during *Hp-WT* and *Hp-ΔcagA* infections. The autophagic flux assay using chloroquine showed that the autophagic index increased significantly in AGS cells pretreated with rapamycin for 24 h (Fig. 4A); furthermore, the autophagic index in AGS cells pretreated with chloroquine for 6 h decreased significantly (Fig. 4B). After 12 h of infection, the protein level of COX-2 decreased significantly in cells infected with *Hp-WT* after treatment with the enhancer. Conversely, there was a slight decrease in the protein level of COX-2 in cells infected with *Hp-ΔcagA*, although the difference was not statistically significant (Fig. 4C). Moreover, the protein level of COX-2 increased in cells infected with *Hp-ΔcagA* after treatment with the inhibitor. Conversely, there was a slight increase in the protein level of COX-2 in cells infected with *Hp-WT* although the difference was not statistically significant (Fig. 4D). These data suggest that autophagy plays a role in COX-2 production induced by *H. pylori.* In addition, autophagic inhibition induced by CagA may represent one of the causative factors associated with the difference in COX-2 production between cells infected with *Hp-WT* and *Hp-ΔcagA*.

### Knockdown of *BECN1* upregulated COX-2 in AGS cells infected with *Hp-ΔcagA*

Finally, to assess the effect of CagA-induced *BECN1* downregulation, AGS cells and *BECN1* knockdown AGS cells were infected with *Hp-WT* and *Hp-ΔcagA*. The autophagic flux assay showed that autophagy was inhibited in AGS cells transfected with *BECN1* si-RNA (Fig. 4E). In *BECN1* knockdown AGS cells infected with *Hp-ΔcagA*, COX-2 expression increased significantly. However, the COX-2 protein level in cells infected with *Hp-WT* decreased slightly, and the difference was not statistically significant (Fig. 4F). Therefore, CagA protein negatively regulates autophagy potentially via the inhibition of BECN1 expression. Moreover, autophagy inhibition induced by CagA may represent a causative factor promoting COX-2 upregulation in AGS cells infected with cagA-positive *H. pylori*.

## Discussion

The role of CagA in autophagy has not been investigated extensively. Therefore, we determined the effect of CagA on autophagy in gastric epithelial cells and the production of an autophagy-regulated pro-inflammatory mediator, COX-2. The present study revealed that *H. pylori* CagA negatively regulated autophagy in gastric cells to upregulate COX-2, an enzyme involved in inflammation and carcinogenesis. Several epidemiological studies have indicated the close relationship between *H. pylori* infection and gastric cancer [19, 20]. While *H. pylori* infection causes severe inflammation and is involved in carcinogenesis, the molecular mechanisms by which *H. pylori* induces gastric cancer development were first evaluated in 2004 when Hatakeyama et al. reported that CagA is associated with gastric carcinogenesis [2].

CagA undergoes tyrosine phosphorylation in gastric epithelial cells, and the phosphorylated CagA induces various cellular responses such as cell proliferation, cell motility, and cell death inhibition. Recently, Li et al. demonstrated that CagA modulates autophagy in host cells [13]. Over the past years, studies have suggested that autophagy plays a role as a specialized immunological effector and regulates innate immunity to activate antimicrobial defense mechanisms [21]. Several bacterial pathogens such as *Mycobacterium tuberculosis* [22] and *Salmonella* [23] have developed the ability to subvert host cell autophagy to establish a persistent infection. Growing evidence shows that *H. pylori* infection interferes with autophagy in host cells [24]. Recently, it was reported that several virulence factors released by *H. pylori* can modulate autophagy [9, 25]. Although existing literature related to autophagic modulation of host cells infected with *H. pylori* suggests that VacA is the main modulator of the autophagic process [7–11], additional virulence factors released by *H. Pylori,* including CagA, have been found to modulate autophagy [13]. In fact, in our experiments in which AGS cells were infected with *Hp-WT* or *Hp-ΔcagA*, a decrease in autophagic index was observed in both *Hp-WT* and *Hp-ΔcagA* infected cells, which may be due to the effect of autophagy inhibitory factors such as VacA. In addition, the autophagic index of AGS cells infected with *Hp-WT* was decreased in comparison with AGS cells infected with *Hp-ΔcagA*. Thus, these findings support the notion that CagA functions as an autophagy inhibitor and modulates the autophagic flux in gastric cells infected with *H pylori*. We assessed autophagic flux using interpretation data from LC3-II immunoblotting because the assessment of the formation and degradation of autophagosomes is one of the most valuable evaluations of autophagic flux. P62 is regarded as one of indicator of autophagic flux. However, the timing of the increase or decrease in P62 does not necessarily coincide with that of LC3. In addition, factors other than autophagy may be involved in the production and decrease of P62. The dysregulation or disruption of autophagy can induce the development of various inflammatory diseases including hepatitis [26], pancreatitis [27], and inflammatory bowel disease [28]. However, the effect of suppression of autophagy caused by *H. pylori* infection on *H. pylori*-induced chronic gastritis remains unclear.

Li et al. (2017) were the first to report that the CagA protein negatively regulates autophagy and promotes inflammation in gastric epithelium cell infected with *H. Pylori*. They reported that CagA downregulates autophagy and activates the c-Met-PI3K/Akt-mTOR signaling pathway, resulting in an increase in IL-8, IL-1β, and TNF-α levels [13]; however, the effect on COX-2 has not been explored. It has been well established that *H. pylori* infection is strongly related to COX-2 expression [18]. A study published in 2020 by Macias-Ceja et al. demonstrated that the pharmacological stimulation of autophagy inhibits COX-2 expression and inflammation in the mucosa of colitis mice [29]. However, the association between COX-2 expression in human gastric cells infected with *H. pylori* and autophagy has not been established. Our study is the first to report that the COX-2 production in host cells infected with *H. pylori* is potentially affected by autophagy. The differences in COX-2 upregulation in *Hp-WT* infected AGS cells pretreated with chloroquine or transfected with BECN1 si RNA were statistically non-significant. However, COX-2 upregulation showed an upward trend as illustrated in Fig. 4D and Fig. 4F. Our hypothesis from the results of a series of experiments is as follows: since autophagy is suppressed to a greater extent in *Hp-WT* infected cells than in *Hp-ΔcagA* infected cells, the level of COX-2 upregulation is not significant when autophagy inhibitors are administered. Further ex vivo studies should determine the effects of this autophagy-induced change in COX-2 expression on long-term chronic inflammation in the human gastric mucosa. In the present study, we found that the CagA protein negatively regulated levels of BECN1, a key molecular regulator of autophagy which is a core component of the class III phosphatidylinositol 3-kinase complex [30]. Previous studies have shown that some intracellular bacteria, such as *Salmonella enteritidis* [31] and *Coxiella burnetii* [32], reduced BECN1 expression and modulated the autophagy of host cells following invasion, but whether BECN1 is downregulated in *H. pylori* infection has not been explored. Previous reports did not clarify the mechanism of BECN1 downregulation through infection with these bacteria. Jiao et al. reported that *Salmonella enteritidis* effector protein AvrA reduced BECN1 protein level through the JNK pathway [31], although BECN1 m-RNA expression level was not changed. Conversely, we demonstrated that CagA suppresses BECN1 mRNA expression, resulting in a decrease in BECN1 protein levels in cells infected with *Hp-WT*. The results imply that the reduction of BECN1 protein downregulation by CagA involved different mechanisms associated with *Salmonella enteritidis* effector protein AvrA. In addition, BECN1 protein not only plays a key role as a regulator of autophagy but is also a tumor suppressor that shows reduced expression in several cancers [33]. In recent years, a relationship between gastric cancer and BECN1 expression has been reported. Zheng et al. reported that BECN1 overexpression suppressed gastric cancer cell growth in mice and BECN1 down-regulation in gastric cancer was associated with gastric carcinogenesis, progression, and poor prognostic prediction in a meta-analytic review of patients with gastric cancer [34]. However, an evaluation of CagA expression was not performed in this report. Huang et al. reported that BECN1 expression is significantly lower in human gastric cancer tissues, particularly in CagA-positive gastric cancer cases [35]. They evaluated the association between CagA-positive *H. pylori* infection and the suppression of autophagy in gastric cancer tissues. However, no direct comparison of autophagic flux in human gastric tissues and gastric cancer was presented in their report. The novelty of our study is highlighted from the CagA-mediated reduction of BECN1 expression and autophagic flux assay in human gastric cancer cells infected with *Hp-WT* and *Hp-ΔcagA* in the in vitro experiments. The present study had several limitations. First, we did not assess the protein levels of COX2 in cells with knocked-down ATG5 and ULK1. Previous in vitro [36] and ex vivo [37] studies reported that chronic *H. pylori* infection reduced ATG5 expression. However, we did not observe significant reduction of ATG5 protein expression in cells infected with *H. pylori* for 12 h. We assume that the 12-h infection time was not sufficient to elicit a significant difference in ATG5 expression. As for ULK1, the long-term effects of ULK1 on human gastric mucosa with chronic *H. pylori* infection are poorly understood. Secondly, in the present study, we could not assess the long-term effects of autophagy modulated by CagA using clinical samples. Further studies are required to elucidate the effect of autophagy in human gastric mucosa infected with *H. pylori*.

## Conclusions

We demonstrated that the *H. pylori* CagA protein downregulates BECN1 expression and negatively regulates autophagy. Autophagy inhibition induced by CagA might be a causative factor promoting COX-2 upregulation in cells infected with cagA-positive *H. pylori*. Understanding the molecular mechanisms by which *H. pylori* infection modulates autophagy would offer novel insights that could facilitate the development of targeted therapy for *H. pylori* infection.

### Supplementary Information

**Additional file 1:** **Figure S1.** Full-length gels images of the immunoblots in Figure 1A, 1B, 1C, and 1D. Black line boxes indicate the immunoblots used if Figure 1A, 1B, 1C, and 1D.

**Additional file 2:** **Figure S2.** Full-length gels images of the blots in Figure 2A and 2B. Red line box indicated the blots use in Figure 2A. Black line boxes indicate the blots used in Figure 2B.

**Additional file 3:** **Figure S3.** Full-length gels images of the immunoblots in Figure 3A. Black line boxes indicated the immunoblots used in Figure 3A.

**Additional file 4:** **Figure S4.** Full-length gels images of the immunoblots Figure 4A, 4B, 4C, 4D, 4E and 4F. Black line boxes indicated the immunoblots used in Figure 4A, 4B, 4C, 4D, 4E and 4F.

**Additional file 5: Supplement Table 1.** PCR cycling conditions.

## Data Availability

The data underlying this article are available from the corresponding author upon reasonable request.

## Abbreviations

VacA: Vacuolating cytotoxin A
CagA: Cytotoxin-associated gene A
LC3: Microtubule-associated proteins 1A/1B light chain 3A
Hp-WT: Wild-type cagA-positive *H. pylori*
Hp-ΔcagA: CagA-knockout *H. pylori*
RGM1: Rat gastric mucosa1
ATG5: Autophagy related 5
BECN1: Beclin1
COX-2: Cyclooxygenase 2
RT-PCR: Reverse transcription-polymerase chain reaction

## References

[1] Jones KR, Whitmire JM, Merrell DS (2010). A tale of two toxins: *Helicobacter pylori* CagA and VacA modulate host pathways that impact disease. Front Microbiol.

[2] Hatakeyama M (2004). Oncogenic mechanisms of the *Helicobacter pylori* CagA protein. Nat Rev Cancer.

[3] Necchi V, Sommi P, Vanoli A, Fiocca R, Ricci V, Solcia E (2017). Natural history of *Helicobacter pylori* VacA toxin in human gastric epithelium in vivo: vacuoles and beyond. Sci Rep.

[4] Mizushima N (2007). Autophagy: process and function. Genes Dev.

[5] Levine B, Kroemer G (2008). Autophagy in the pathogenesis of disease. Cell.

[6] Mizushima N, Levine B, Cuervo AM, Klionsky DJ. Autophagy fights disease through cellular self-digestion. Nature. 2008;451(1069–75). 10.1038/nature06639. 10.1038/nature06639 PMC2670399 18305538

[7] Ricci V (2016). Relationship between VacA toxin and host cell autophagy in *Helicobacter pylori* infection of the human stomach: a few answers, many questions. Toxins (Basel).

[8] Terebiznik MR, Raju D, Vázquez CL, Torbricki K, Kulkarni R, Blanke SR (2009). Effect of *Helicobacter pylori* vacuolating cytotoxin on the autophagy pathway in gastric epithelial cells. Autophagy.

[9] Raju D, Hussey S, Ang M, Terebiznik MR, Sibony M, Galindo-Mata E (2012). Vacuolating cytotoxin and variants in Atg16L1 that disrupt autophagy promote *Helicobacter pylori* infection in humans. Gastroenterology.

[10] Tsugawa H, Suzuki H, Saya H, Hatakeyama M, Hirayama T, Hirata K (2012). Reactive oxygen species-induced autophagic degradation of *Helicobacter pylori* CagA is specifically suppressed in cancer stem-like cells. Cell Host Microbe.

[11] Tsugawa H, Mori H, Matsuzaki J, Sato A, Saito Y, Imoto M (2019). CAPZA1 determines the risk of gastric carcinogenesis by inhibiting *Helicobacter pylori* CagA-degraded autophagy. Autophagy.

[12] Qian HR, Yang Y (2016). Functional role of autophagy in gastric cancer. Oncotarget.

[13] Li N, Tang B, Jia YP, Zhu P, Zhuang Y, Fang Y (2017). *Helicobacter pylori* CagA protein negatively regulates autophagy and promotes inflammatory response via c-Met-PI3K/Akt-mTOR signaling pathway. Front Cell Infect Microbiol.

[14] Asahi M, Azuma T, Ito S, Ito Y, Suto H, Nagai Y (2000). *Helicobacter pylori* CagA protein can be tyrosine phosphorylated in gastric epithelial cells. J Exp Med.

[15] Suzuki M, Mimuro H, Kiga K, Fukumatsu M, Ishijima N, Morikawa H (2009). *Helicobacter pylori* CagA phosphorylation-independent function in epithelial proliferation and inflammation. Cell Host Microbe.

[16] Hayashi Y, Tsujii M, Wang J, Kondo J, Akasaka T, Jin Y (2013). CagA mediates epigenetic regulation to attenuate let-7 expression in *Helicobacter pylori*-related carcinogenesis. Gut.

[17] Inoue T, Hayashi Y, Tsujii Y, Yoshii S, Sakatani A, Kimura K (2021). Suppression of autophagy promotes fibroblast activation in p53-deficient colorectal cancer cells. Sci Rep.

[18] Cheng J, Fan XM (2013). Role of cyclooxygenase-2 in gastric cancer development and progression. World J Gastroenterol.

[19] Murakami K, Kodama M, Fujioka T (2006). Latest insights into the effects of *Helicobacter pylori* infection on gastric carcinogenesis. World J Gastroenterol.

[20] Cao Y, Luo Y, Zou J, Ouyang J, Cai Z, Zeng X (2019). Autophagy and its role in gastric cancer. Clin Chim Acta.

[21] Yuk JM, Yoshimori T, Jo EK (2012). Autophagy and bacterial infectious diseases. Exp Mol Med.

[22] Zhai W, Wu F, Zhang Y, Fu Y, Liu Z (2019). The immune escape mechanisms of *Mycobacterium tuberculosis*. Int J Mol Sci.

[23] Wu S, Shen Y, Zhang S, Xiao Y, Shi S (2020). *Salmonella* interacts with autophagy to offense or defense. Front Microbiol.

[24] Deen NS, Gong L, Naderer T, Devenish RJ, Kwok T (2015). Analysis of the relative contribution of Phagocytosis, LC3-associated Phagocytosis, and Canonical autophagy during *Helicobacter pylori* infection of macrophages. Helicobacter.

[25] Bravo J, Díaz P, Corvalán AH, Quest AFG (2019). A novel role for *Helicobacter pylori* Gamma-Glutamyltranspeptidase in regulating autophagy and bacterial internalization in human gastric cells. Cancers (Basel).

[26] Zhang L (2020). Autophagy in hepatitis B or C virus infection: An incubator and a potential therapeutic target. Life Sci.

[27] Gukovskaya AS, Gukovsky J, Algül H, Habtezion A (2017). Autophagy, inflammation, and immune dysfunction in the pathogenesis of pancreatitis. Gastroenterology.

[28] Hampe J, Franke A, Rosenstiel P, Till A, Teuber M, Huse K (2007). A genome-wide association scan of nonsynonymous SNPs identifies a susceptibility variant for Crohn disease in ATG16L1. Nat Genet.

[29] Macias-Ceja DC, Cosín-Roger J, Ortiz-Masiá D, Salvador P, Hernández C, Esplugues JV (2017). Stimulation of autophagy prevents intestinal mucosal inflammation and ameliorates murine colitis. Br J Pharmacol.

[30] McKnight NC, Zhenyu Y (2013). Beclin 1, an Essential component and master regulator of PI3K-III in health and disease. Curr Pathobiol Rep.

[31] Jiao Y, Zhang YG, Lin Z, Lu R, Xia Y, Meng C (2020). *Salmonella enteritidis* effector AvrA suppresses autophagy by reducing Beclin-1 protein. Front Immunol.

[32] Vázquez CL, Colombo MI (2010). Coxiella burnetii modulates Beclin 1 and Bcl-2, preventing host cell apoptosis to generate a persistent bacterial infection. Cell Death Differ.

[33] Aita VM, Liang XH, Murty VV, Pincus DL, Yu W, Cayanis E (1999). Cloning and genomic organization of beclin 1, a candidate tumor suppressor gene on chromosome 17q21. Genomics.

[34] Zheng H, Zhao S, Xue H, Zhao E, Jiang H, Hao C (2020). The roles of Beclin 1 expression in gastric cancer: a marker for carcinogenesis, aggressive behaviors and favorable prognosis, and a target of gene therapy. Front Oncol.

[35] Huang X, Wang C, Sun J, Luo J, You J, Liao L (2018). Clinical value of CagA, c-Met, PI3K and Beclin-1 expressed in gastric cancer and their association with prognosis. Oncol Lett.

[36] Yang XJ, Si RH, Liang YH, Ma BQ, Jiang ZB, Wang B (2016). Mir-30d increases intracellular survival of Helicobacter pylori through inhibition of autophagy pathway. World J Gastroenterol.

[37] Tanaka S, Nagashima H, Uotani T, Graham DY, Yamaoka Y (2017). Autophagy-related genes in Helicobacter pylori infection. Helicobacter.

