# Supplementary figures and images for: A novel role for Helicobacter pylori cytotoxin-associated gene A in negative regulation of autophagy in human gastric cells
Source: BMC Gastroenterol. 2023 Sep 22;23:326. doi: 10.1186/s12876-023-02944-8 (PMC10517455; doi:10.1186/s12876-023-02944-8)

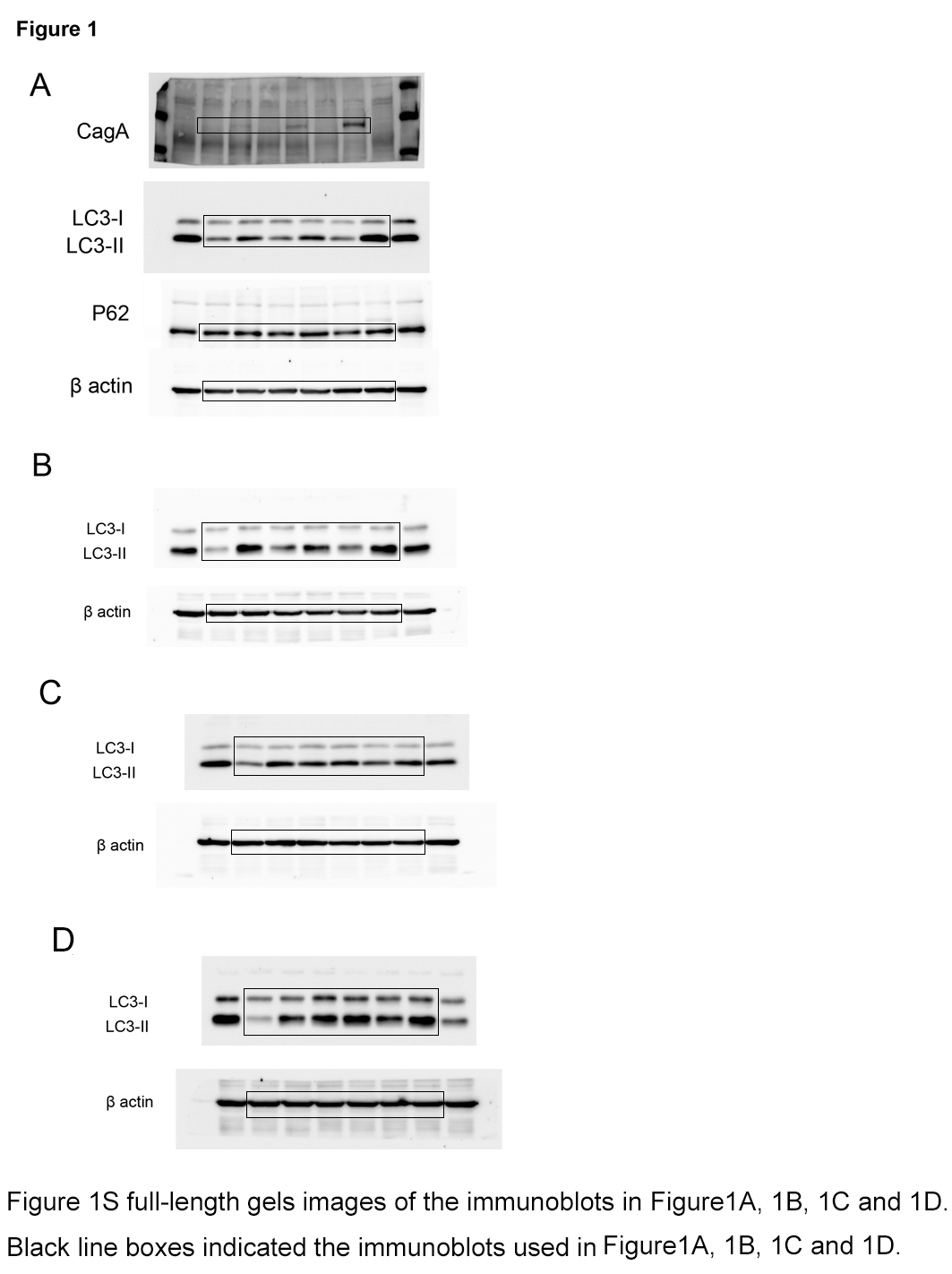

Supplement: Supplementary file 1 — Additional file 1: Figure S1. Full-length gels images of the immunoblots in Figure 1A, 1B, 1C, and 1D. Black line boxes indicate the immunoblots used if Figure 1A, 1B, 1C, and 1D. [file 12876_2023_2944_MOESM1_ESM.tif]

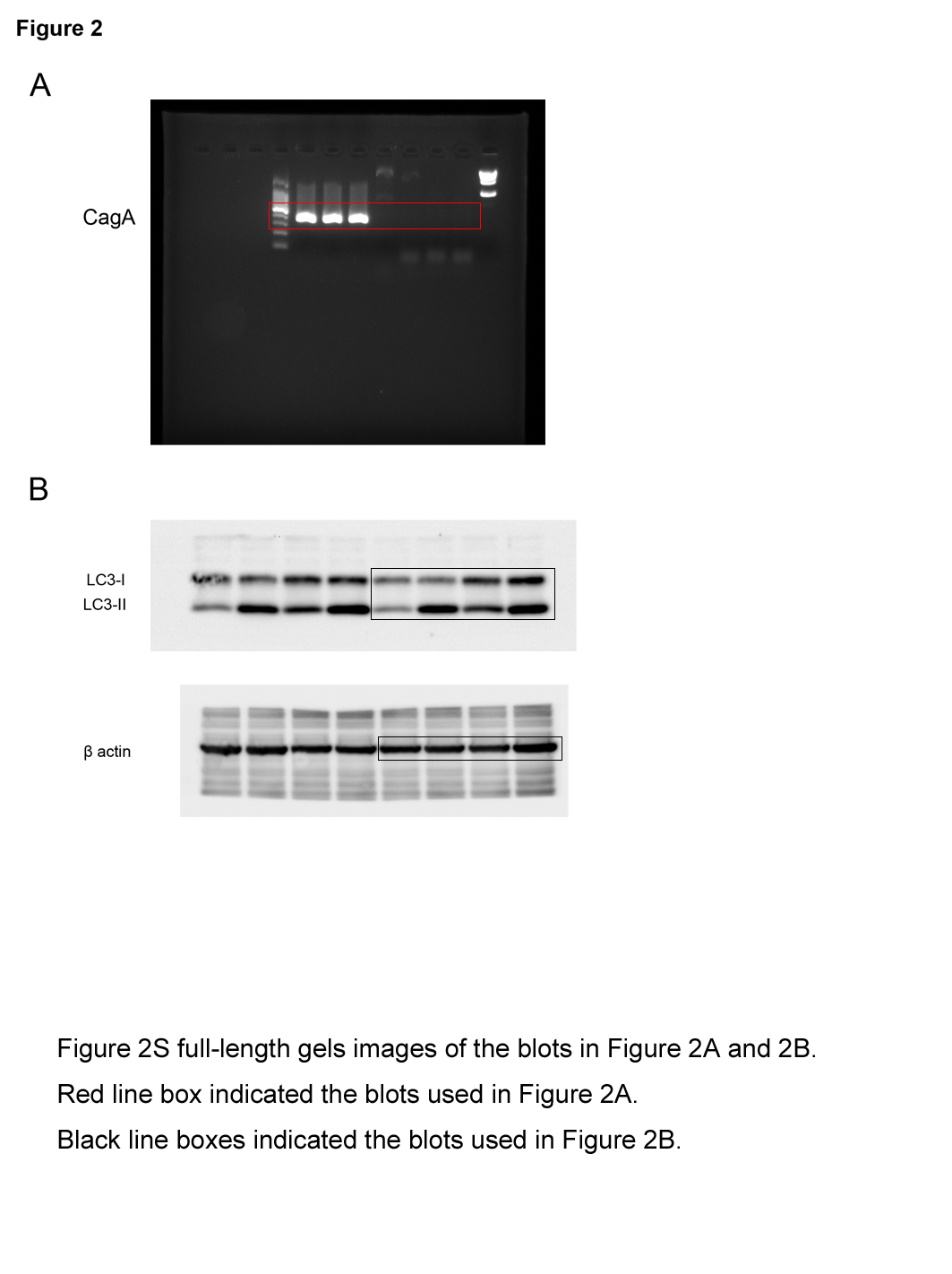

Supplement: Supplementary file 2 — Additional file 2: Figure S2. Full-length gels images of the blots in Figure 2A and 2B. Red line box indicated the blots use in Figure 2A. Black line boxes indicate the blots used in Figure 2B. [file 12876_2023_2944_MOESM2_ESM.tif]

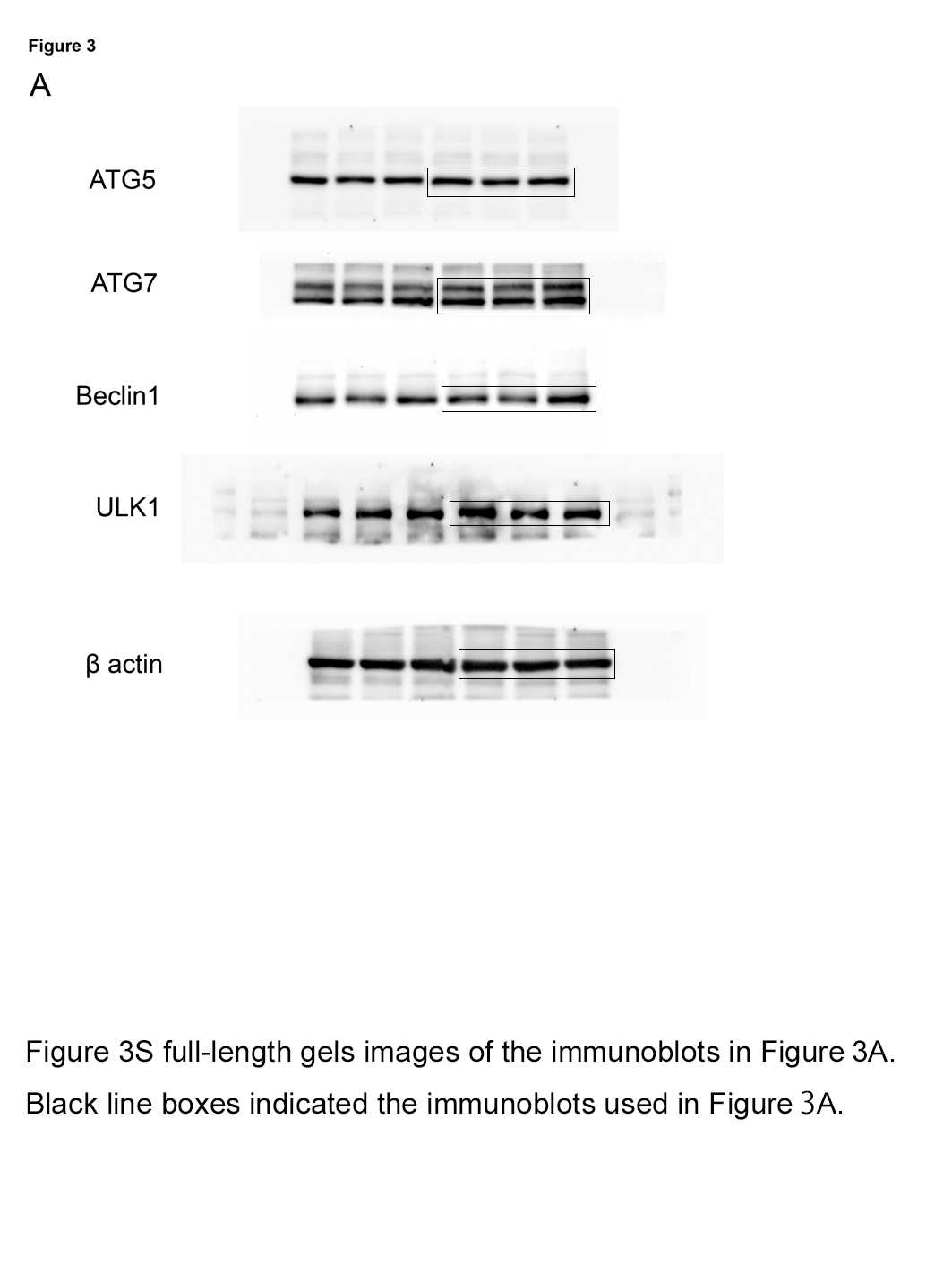

Supplement: Supplementary file 3 — Additional file 3: Figure S3. Full-length gels images of the immunoblots in Figure 3A. Black line boxes indicated the immunoblots used in Figure 3A. [file 12876_2023_2944_MOESM3_ESM.tif]

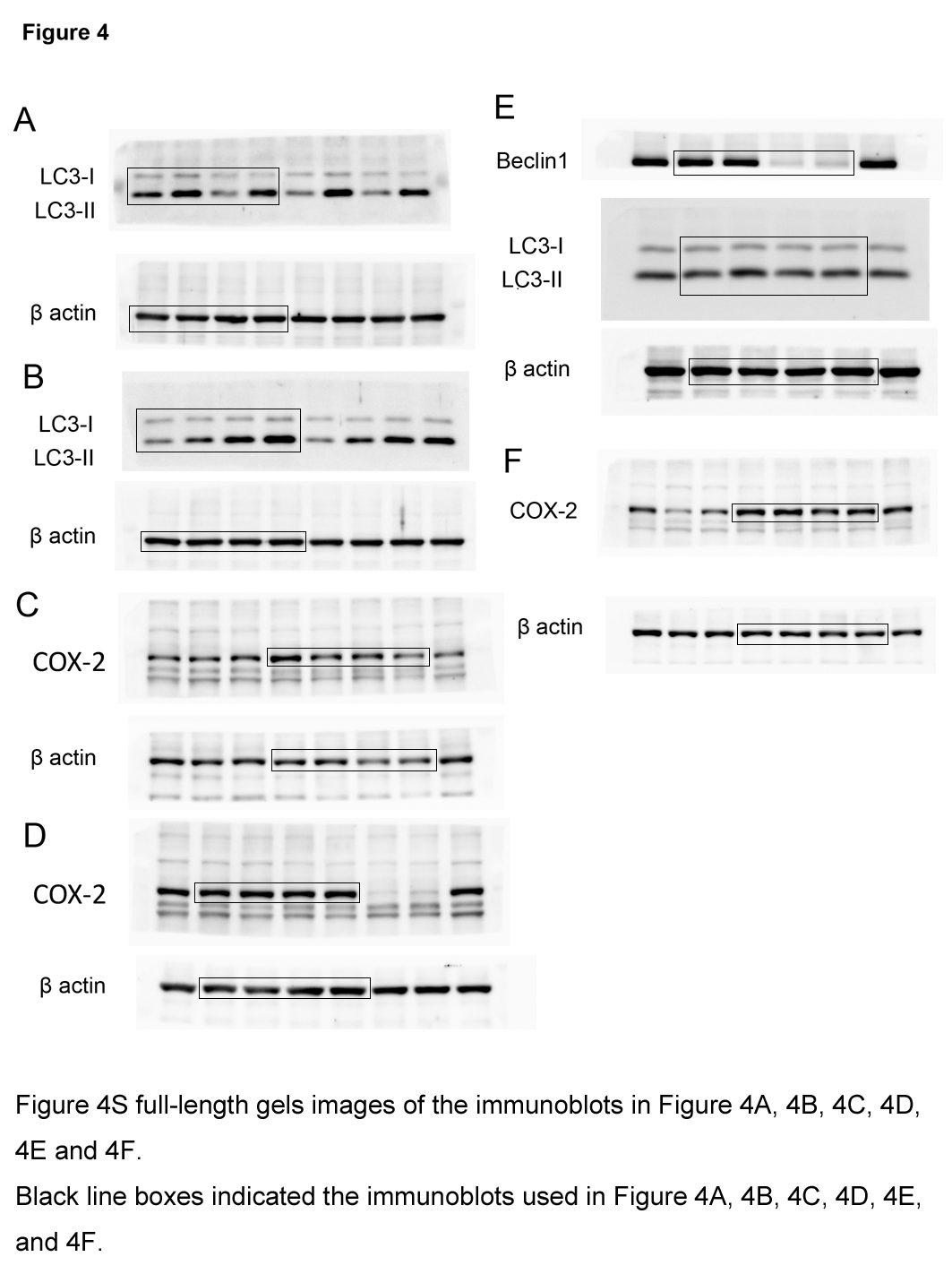

Supplement: Supplementary file 4 — Additional file 4: Figure S4. Full-length gels images of the immunoblots Figure 4A, 4B, 4C, 4D, 4E and 4F. Black line boxes indicated the immunoblots used in Figure 4A, 4B, 4C, 4D, 4E and 4F. [file 12876_2023_2944_MOESM4_ESM.tif]
